# Diffuse plane xanthomas as the first manifestation of multiple myeloma

**DOI:** 10.1111/ddg.15807

**Published:** 2025-06-12

**Authors:** Eleni Koutra, Elke Lusmöller, Hosam Ghanem, Astrid Aumann, Jens Atzpodien, Rudolf Stadler

**Affiliations:** ^1^ Department of Dermatology Venereology Allergology and Phlebology Johannes Wesling Klinikum Minden Germany; ^2^ Center for Hematology and Oncology Niels Stensen Kliniken Franziskus Hospital Harderberg Georgsmarienhütte Germany

Dear Editors,

Diffuse plane xanthomatosis is a rare non‐Langerhans cell histiocytosis characterized by clonal proliferation of abnormal histiocytic cells with intracellular lipid accumulation. Clinical features include xanthelasma palpebrarum and yellow macules or patches symmetrically distributed in the periorbital region, on the neck, submammary areas, upper trunk as well as flexural regions of the axillae and groin. These lesions are typically asymptomatic, aside from occasional.Although classified among the normolipidemic xanthomas as first described by Altman and Winkelmann in 1962,^1^, ^2^ cases associated with type II or IV hyperlipoproteinemia according to Fredrickson's classification have occasionally been reported.^3^, ^4^ We report the case of a patient with diffuse plane xanthomas as the first clinical manifestation of multiple myeloma, successfully treated in collaboration with hematologists.

A 61‐year‐old woman presented for outpatient evaluation of gradually progressive yellowing of the skin over two years. She described yellow streaks that had increased in size over time, occasionally accompanied by itching and burning. On clinical examination, symmetrically distributed yellow macules were noted in the periorbital, axillary, submammary andinguinal regions (Figure 1a,b). There were no B symptoms or lymphadenopathy. Her medical history included a well‐controlled arterial hypertension. Blood tests revealed an elevated erythrocyte sedimentation rate and type IIa hypercholesterolemia with an LDL cholesterol level of 135 mg/dl (reference range: LDL < 116 mg/dl). Serum electrophoresis indicated monoclonal gammopathy with increased free kappa light chains. Vitamin A serology was normal.Differential diagnoses of yellow skin discoloration include xanthelasma, jaundice, carotenemia, drug‐induces xanthoderma (from mepacrine or sorafenib), phenol exposure and chrysiasis.

**FIGURE 1:**
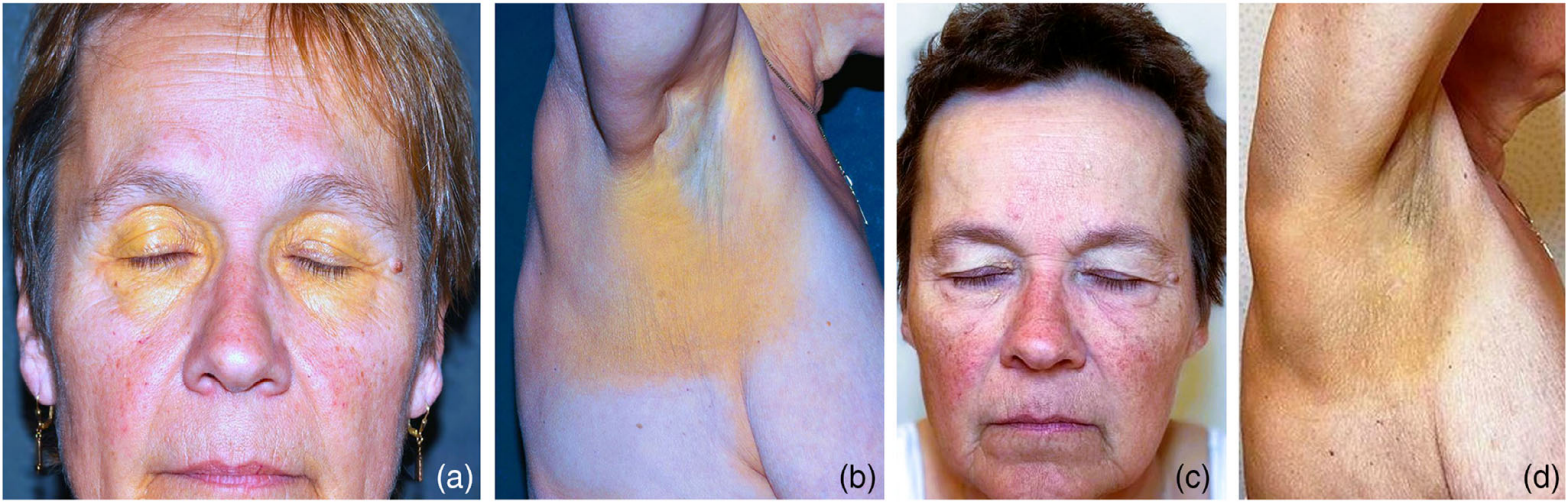
(a, b) Clinical presentation: Periorbital, cervical, axial and submammary maculae. (c, d) Significant improvement after immunochemotherapy and autologous stem cell transplantation.

Histopathologic examination of a hematoxylin‐eosin‐stained lesional skin biopsy from the right axilla (Figure 2a) revealed a diffuse dermal infiltration of lipid‐laden macrophages, foam cells, arranged in a band‐like pattern throughout the reticular dermis, confirmed by CD68 staining (Figure 2b). The lipid staining demonstrated intracellular lipid accumulation (Figure 2c). Furthermore, Langerhans cell histiocytosis was ruled out via CD1a and Langerin CD207 staining. In the light chain restriction staining, Kappa was diffusely positive in the affected macrophages (Figure 2d).

**FIGURE 2:**
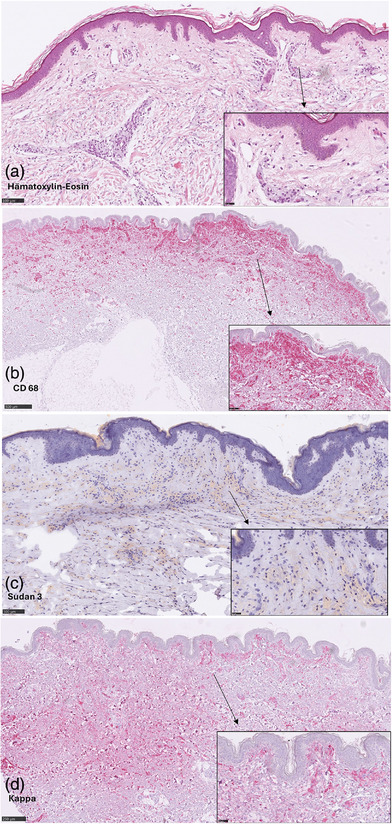
(a) Diffuse foam cells infiltration with round hyperchromatic nuclei and wide cytoplasm. (b) CD68 staining shows band‐like macrophages distribution in thereticular dermis. (c) The lipid staining showed intracellular lipid deposition. (d) Light chain restriction staining shows kappa positivity in the affected macrophages (d).

Based on these findings, we hypothesized a paraneoplastic association betwenn the xanthomas and multiple myeloma and referred the patient for further hematological evaluation close to home. Initially, due to the hypercholesterolemia, a conventional statin therapy was initiated after an endocrinological assessment. Subsequently our suspicion was confirmed with a bone marrow biopsy demostrating 85% plasma cell infiltration. Whole body computed tomography showed no osteolytic lesions. After multiple oncological therapies including immunochemotherapy and autologous stem cell transplantation, the paraproteinemia and hypercholesterolemia normalized and the cutaneous lesions significantly improved (Figure 1c,d).

## DISCUSSION

The immunological and pathogenic role of paraproteinemia in lipoprotein disorders and plane xanthoma formation remains unclear.^3^, ^4^ It is postulated that monoclonal immunoglobulins bind to circulating low‐density plasma lipoproteins, forming complexes that deposit perivascularly in the skin. These complexes are phagocytosed by macrophages, which then differentiate into foam cells.^3^, ^5^, ^6^ In 1966, Lynch and Winkelmann reported a strong association between diffuse plane xanthomas and disorders of the reticuloendothelial system.^2^ In large case series, multiple myeloma was present in 48% of patients, and monoclonal gammopathy of undetermined significance in 41%.^5^, ^6^, ^7^, ^8^, ^9^ Other associations include leukemia, malignant lymphoma including mycosis fungoides and Sézary syndrome, Castleman disease and cryoglobulinemia.^10^ Typically, cutaneous manifestations precede the hematologic diagnoses by several years, emphasizing the need for long‐term monitoring. The prognosis depends on the underlying disease, and should guide targeted treatment strategies.

Our case highlights the importance of evaluating yellow skin discoloration for systemic associations. Dermatologists play a critical role in the early recognition of potentially serious systemic diseases requiring prompt treatment.

## CONFLICT OF INTEREST STATEMENT

None.
